# Modelling direct healthcare costs for screening and treatment of retinopathy of prematurity among infants born at gestational age <24 weeks: findings from a Swedish cohort

**DOI:** 10.1136/bmjophth-2025-002507

**Published:** 2025-12-31

**Authors:** Chatarina Löfqvist, Shambhavi Sharma, Ann Hellström, Hanna Gyllensten

**Affiliations:** 1 Institute of Health and Care Sciences, Sahlgrenska Academy, University of Gothenburg, Gothenburg, Sweden; 2 Department of Clinical Neuroscience, Institute of Neuroscience and Physiology, Sahlgrenska Academy, University of Gothenburg, Gothenburg, Sweden; 3 University of Gothenburg Centre for Person-centred Care (GPCC), Sahlgrenska Academy, University of Gothenburg, Gothenburg, Sweden

**Keywords:** Treatment Lasers, Treatment Medical, Child health (paediatrics), Retina, Treatment Expectations

## Abstract

**Objective:**

Sweden has a long-standing tradition of actively managing infants born extremely preterm at 22–23 weeks’ gestational age. This study analyses screening and treatment pathways for retinopathy of prematurity (ROP) in these infants, assessing costs and health outcomes to develop a model of direct healthcare costs.

**Method and analysis:**

The cohort included all 399 infants born at 22–23 weeks in Sweden (2007–2018) who underwent ROP screening, recorded in the national ROP registry SWEDROP. A health economic model estimated costs based on three primary pathways: (1) no sight-saving treatment, (2) laser as initial treatment and (3) anti-vascular endothelial growth factor (anti-VEGF) as initial treatment. Pathways 2 and 3 were further divided into single and multiple treatments. Costs were calculated using screening frequency, treatment and neonatal care expenses. Register data were verified against medical records. An expanded model incorporated gestational age and comorbidities.

**Results:**

In the basic model, 36% received laser (16 screenings on average; 32% required retreatment), while 7% received anti-VEGF injections (25 screenings; 69% required retreatment). The cost of screening and treating an infant with laser was Int$ 18 590, compared with Int$ 20 792 for anti-VEGF. The expanded models showed similar screening and treatment frequencies.

**Conclusion:**

Despite similar overall costs, the higher screening burden in the anti-VEGF group (25 vs 16 screenings) raises concerns regarding cost-effectiveness and potential health impacts. Main limitations include the use of cost data from a single hospital, potential selection bias between treatment groups and limited precision in small subgroups. These findings lay the groundwork for future research on long-term health and cost outcomes in this vulnerable population.

**WHAT IS ALREADY KNOWN ON THIS TOPIC**

- Laser and anti-vascular endothelial growth factor (anti-VEGF) are established treatments for retinopathy of prematurity (ROP), but comparative cost and screening burden data from real-world cohorts are limited.
- Existing health-economic models in ROP have largely focused on screening strategies rather than treatment choice.

**WHAT THIS STUDY ADDS**

- In this national cohort of infants born at 22–23 weeks’ gestation, anti-VEGF was associated with substantially more screenings and retreatments than laser.
- Despite the lower per-procedure cost of anti-VEGF, overall costs were slightly higher compared with laser.
- A small subgroup (infants with aggressive posterior ROP) showed potential clinical equipoise in which anti-VEGF was cost-effective in the model.

**HOW THIS STUDY MIGHT AFFECT RESEARCH, PRACTICE OR POLICY**

- The study illustrates how observational data can be used to map real-world ROP screening and treatment pathways.
- The findings may help identify subgroups suitable for future comparative effectiveness research.
- The decision-tree structure could support more refined modelling in future studies.

## Introduction

 Retinopathy of prematurity (ROP) is a leading cause of childhood blindness globally and a major complication of extreme preterm birth, alongside other severe morbidities such as bronchopulmonary dysplasia, intraventricular haemorrhage (IVH) and necrotising enterocolitis (NEC).^1^ Disruption of intrauterine retinal neuronal and vascular development results in abnormal retinal vascularisation, which, depending on severity, may require follow-up and treatment.^2 3^

According to national guidelines, all infants born at a gestational age below a specified threshold undergo ROP screening,^4 5^ followed by treatment with either laser photocoagulation or anti-vascular endothelial growth factor (anti-VEGF) injections when indicated. However, the frequency of screening and its associated costs in relation to treatment choice remain largely unexplored. While randomised controlled trials are the gold standard for comparing treatments, in situations where cost-effectiveness estimates from such trials are lacking, health-economic models can also be used to simulate cost-effectiveness using observational real-world data, findings from the literature, expert opinions and assumptions.^6^ Decision-tree models are simple health economic models that can be used to explore and illustrate decisions and alternative treatment pathways. Such models have been used in the field of ROP before to explore alternative screening programmes and the timing and choice of treatment.^7^ There have also been modelling studies exploring recurrence after treatment.^8^ To our knowledge, no health economic modelling study explores the clinical pathways associated with the decision between initiating ROP treatment using anti-VEGF or laser. There are RCTs examining the effectiveness of anti-VEGF and laser, suggesting beneficial outcomes of anti-VEGF, particularly for posterior disease.^9 10^ A network meta-analysis found lower retreatment rates with laser than with ranibizumab overall, while bevacizumab was associated with the most durable response in Zone I ROP.^11^ However, to develop a useful health-economic model for clinical decision-making using available observational data, where initiation with either anti-VEGF or laser is a valid choice, the infants in each treatment pathway should be limited to those who could have received either treatment first, ie, those with clinical equipoise.

To identify subgroups of infants in which the choice between treatment initiation with either laser or anti-VEGF is a valid choice, we describe clinical pathways for ROP screening and treatment among infants born at 22–23 weeks’ gestation in Sweden and map cost distributions associated with ROP screening and treatment. This study aimed to describe the real-world clinical pathways and cost patterns for ROP treatment among extremely preterm infants in Sweden and to develop a stepwise, adaptable decision tree model for assessing treatment choices and resource utilisation. By gradually incorporating clinical complexity, the model allows exploration of subgroups where clinical equipoise may support comparative evaluation.

## Methods

This study is based on clinical data from TINY, a retrospective register-based study following all infants born extremely preterm in Sweden.^12 13^ This reporting follows the Consolidated Health Economic Evaluation Reporting Standards.^14^

### Study design

The study employed a decision tree framework to explore the cost-effectiveness of alternative treatment pathways for ROP. Decision-tree models are graphical tools that map clinical decisions and possible outcomes, assigning probabilities, costs and health effects to each branch. They are especially suited for modelling acute, one-time decisions, such as the initial choice between anti-VEGF and laser treatment in ROP, and are widely used in health economic evaluations to compare competing interventions^15 16^

### Study population

The study population included all infants born before 24 weeks’ gestation in Sweden (2007–2018), from the initial hospitalisation through their final ROP screening (up to 1 year of age). Infants were identified from SWEDROP, the Swedish National ROP registry,^5^ which covers all infants screened for ROP as part of the national programme.

Sweden’s universal healthcare system,^17^ funded primarily through taxation, provides free care for infants, including advanced neonatal and ophthalmological care. Extremely preterm infants receive specialised neonatal intensive care at selected university hospitals, with follow-up and additional care provided at regional hospitals.^17^

### Data sources and variables

Personal identification numbers enabled the linkage of records across registers and medical records. Model parameters included probabilities and health outcomes derived from individual-level data, including the following data sources:

- SWEDROP (part of the SNQ clinical register), held by Uppsala University Hospital, for screening and treatment data.
- Medical chart/record review for data validation.

### Health economic model parameters

Model payoffs were cost estimates from a healthcare perspective, including screening and treatment costs (table 1) at a regional hospital in Gothenburg, Sweden. All costs were inflated to 2022 values, adjusted for salary and healthcare price changes (excluding medications) and converted to international dollars (Int$) using purchasing power parities. Costs were not discounted due to the short time horizon (at most around 50 weeks according to clinical guidelines).

**Table 1:** Unit costs and source of cost data of included resources

Resource	Unit cost from source	Unit cost (2022 value)*	Source
NICU cost (per day)	SEK 30 000 (2021 value) = SEK 31 110 in 2022	Int$ 3704	Based on an economic assessment in a report from the National Board of Health and Welfare^22^
ROP screening/examination (per session)	SEK 3553 (2021 value) = SEK 3 684 in 2022	Int$ 439	Average cost for KVÅ AC037 from the Sahlgrenska University Hospital administrative register
LASER treatment in the operating theatre (per session)	SEK 70 000 (2022 value)	Int$ 8333	Approximation
Anti-VEGF Injections in the operating theatre (per session)	1/3 of SEK 70 000 +SEK 3100 = SEK 26 433 (2022 value)	Int$ 3147	Approximation for resource use in surgery during the session. Cost for Lucentis injection from the Sahlgrenska University Hospital administrative register

* The conversion was conducted using an index for local salary and price change in healthcare, adjusted for quality change but excluding medications (3.7% increase from 2021 to 2022)^23^ and thereafter converted using purchasing power parities^24^ from SEK to Int$ (conversion rate in 2022: SEK 8.4=Int$ 1), according to recommended methodology for non-tradable resources.^25^

NICU, neonatal intensive care unit; ROP, retinopathy of prematurity.

Health outcomes included vision impairment at follow-up. Visual impairment was defined as referral to a low-vision clinic and/or visual acuity <20/60 at ≥3.5 years, consistent with our cohort follow-up.^13^ As the model focuses on acute-phase management, visual impairment was not included as a cost input. Neonatal intensive care unit (NICU) length of stay was reported descriptively but excluded from the decision tree as it is not directly attributable to ROP screening or treatment.

### Decision tree model

The decision tree was constructed using TreeAge Pro Healthcare 2024 R1.0. Infants were initially categorised, in steps, based on:

1. ROP treatment or no treatment.
2. Initial treatment type (laser or anti-VEGF).
3. Presence of early comorbidities (NEC, IVH 3–4, periventricular leukomalacia).
4. Gestational age at birth.

The ‘no treatment’ branch reflects infants who, in observed clinical practice, did not meet criteria for ROP treatment; no functional consequences or costs were imputed. Thus, the initial model was based on whether infants received treatment. Thereafter, models were built in steps, incorporating each factor. The final model included decision nodes^18^ for treatment choice and gestational age, supporting future investigations into the cost-effectiveness of delaying preterm delivery. Comparisons after each model iteration identified remaining differences between groups, limiting the possibility of identifying a division indicating equipoise, which could be used as a decision node. Thereafter, a data-driven approach identified aggressive posterior (AP) ROP as a key factor influencing treatment variation between study sites, and it was subsequently incorporated as a grouping variable in the model.

### Analysis

Infants were stratified into two gestational age groups: infants born at 21+6–22+6 (weeks+days) and 23+0–23+6. Descriptive statistics were generated using Stata 18.5 (StataCorp LLC). Patient characteristics were summarised using frequencies, means and SD. Resource use and costs were reported as means with 95% CIs. Folding back the decision tree provided an average cost per infant from the healthcare perspective. The final model was visualised using a cost-effectiveness plane.

### Ethics

The study was approved by the Swedish Ethical Review Authority (DNR 2019–05265, DNR 2021-06161-02).

## Results

The study used data on 399 infants born extremely preterm and surviving to 40 weeks postmenstrual age; 225 infants (56%) had no ROP treatment, while 145 (36%) and 29 (7%) had laser or anti-VEGF as their first ROP treatment, respectively. The use of anti-VEGF as first-line treatment increased over the study period (2007–2018), consistent with national practice changes. Severe early comorbidities (NEC requiring surgery and/or IVH grade 3–4) occurred among 112 infants (28.1%) (table 2).

**Table 2:** Descriptive statistics of infants by ROP treatment

	No ROP treatment		Laser first ROP treatment		Anti-VEGF first ROP treatment		Total	
	N	%	N	%	N	%	N	%
	225	100.0%	145	100.0%	29	100.0%	399	100.0%
GA group								
22 w	50	22.2%	33	22.8%	15	51.7%	98	24.6%
23 w	175	77.8%	112	77.2%	14	48.3%	301	75.4%
Twin								
No twin	193	86.2%	116	80.0%	25	86.2%	334	83.9%
Twin	31	13.8%	29	20.0%	4	13.8%	64	16.1%
Severe early comorbidities (NEC, IVH)								
No	172	76.4%	98	67.6%	17	58.6%	287	71.9%
Yes	53	23.6%	47	32.4%	12	41.4%	112	28.1%
Region								
Uppsala	57	25.3%	27	18.6%	13	44.8%	97	24.3%
Göteborg	30	13.3%	38	26.2%	2	6.9%	70	17.5%
Linköping	15	6.7%	13	9.0%	2	6.9%	30	7.5%
Lund	39	17.3%	20	13.8%	0	0.0%	59	14.8%
St Erik	34	15.1%	24	16.6%	10	34.5%	68	17.0%
Umeå	39	17.3%	19	13.1%	1	3.4%	59	14.8%
Örebro	11	4.9%	4	2.8%	1	3.4%	16	4.0%

Anti-VEGF, anti-vascular endothelial growth factor; GA, gestational age (at birth); IVH, intraventricular haemorrhage; NEC, necrotising enterocolitis; ROP, retinopathy of prematurity.

### Preplan modelling

A first step in building the model was to identify the treated and untreated groups (online supplemental figure 1A). As expected, screening and treatment costs were higher among infants treated for ROP. They also had more screening visits when compared with those who did not require treatment (with treatment 17.6 (16.6–18.6) vs without treatment 13.3 (12.5–14.2) (online supplemental table 1).

Thereafter, pathways were divided for infants with ROP, with treatment initiated using either anti-VEGF or laser (online supplemental figure 1B). Comparing the treatment groups, the anti-VEGF group drives a higher number of screenings (number at anti-VEGF: 25.0 (23.0–26.9) vs laser 16.1 (15.1–17.1)), with laser accounting for only 64% of the anti-VEGF group screenings, and more often needed retreatment (69% vs 32%). In Sweden, many extremely preterm infants (22–23 weeks GA) undergo twice-weekly screening before treatment, which substantially increases the total number of examinations. Several infants treated with laser also developed treatment-warranted ROP comparatively late, extending screening far beyond what is expected in trial-based protocols. This pattern is consistent with national data showing prolonged disease courses and variation between centres.^12^ NICU days were also longer for the anti-VEGF group (180.1 days vs 150.9 days), although the 95% CI overlapped (online supplemental table 1). Therefore, costs were higher among infants with ROP who were initially treated with anti-VEGF, even if the cost of the anti-VEGF treatment itself is lower than the cost of laser treatment. The cost of screening and treating an infant with laser was Int$ 18 590, compared with Int$ 20 792 for anti-VEGF (online supplemental table 1). However, it appears that this difference between groups is also influenced by something other than the initial treatment choice, as these infants also had more extended stays in the NICU. This indicates that the groups are not directly comparable based solely on treatment type.

Adding the occurrence of early comorbidities (online supplemental figure 1C) could explain the identified difference in NICU stay. Screening and treatment costs also remained higher in the group initiating treatment with anti-VEGF. However, they were very similar between infants with and without early comorbidities, thus still not indicating clinical equipoise.

When including the gestational age (online supplemental figure 1D), this revealed a bias in the distribution of infants between pathways, where costs were suddenly higher among infants born at 22 weeks’ gestational age with comorbidities and those born at 23 weeks without comorbidities. However, all pathways still indicated that costs for screening and treatment were higher among infants treated first with anti-VEGF, which contradicted the expectations for pathways in a state of clinical equipoise.

### Data-driven modelling

**Figure 1:**
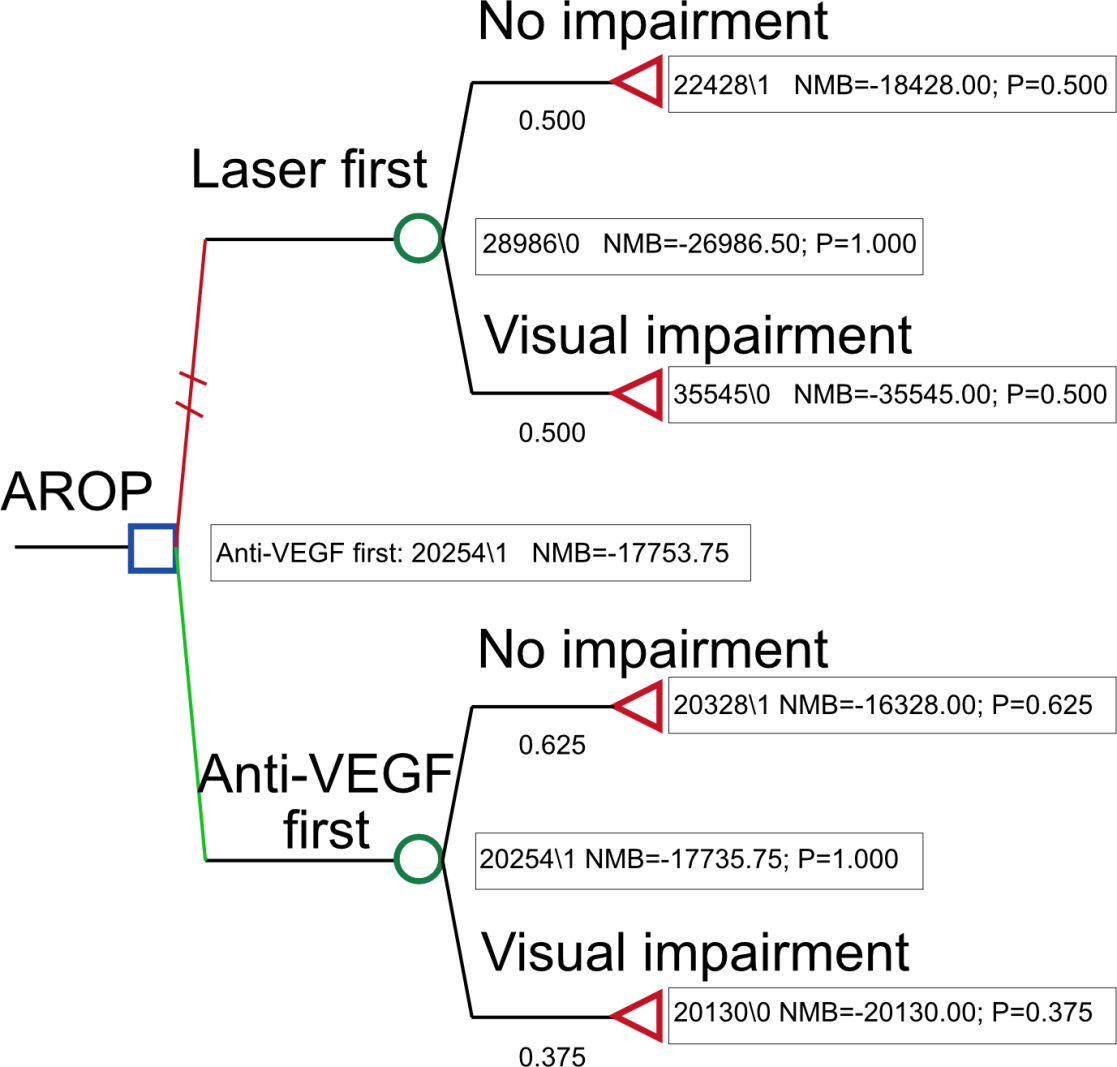
Decision tree for treatment pathways among infants with AROP. Anti-VEGF, anti-vascular endothelial growth factor; AROP, aggressive posterior retinopathy of prematurity; NMB, net monetary benefit.

**Figure 2:**
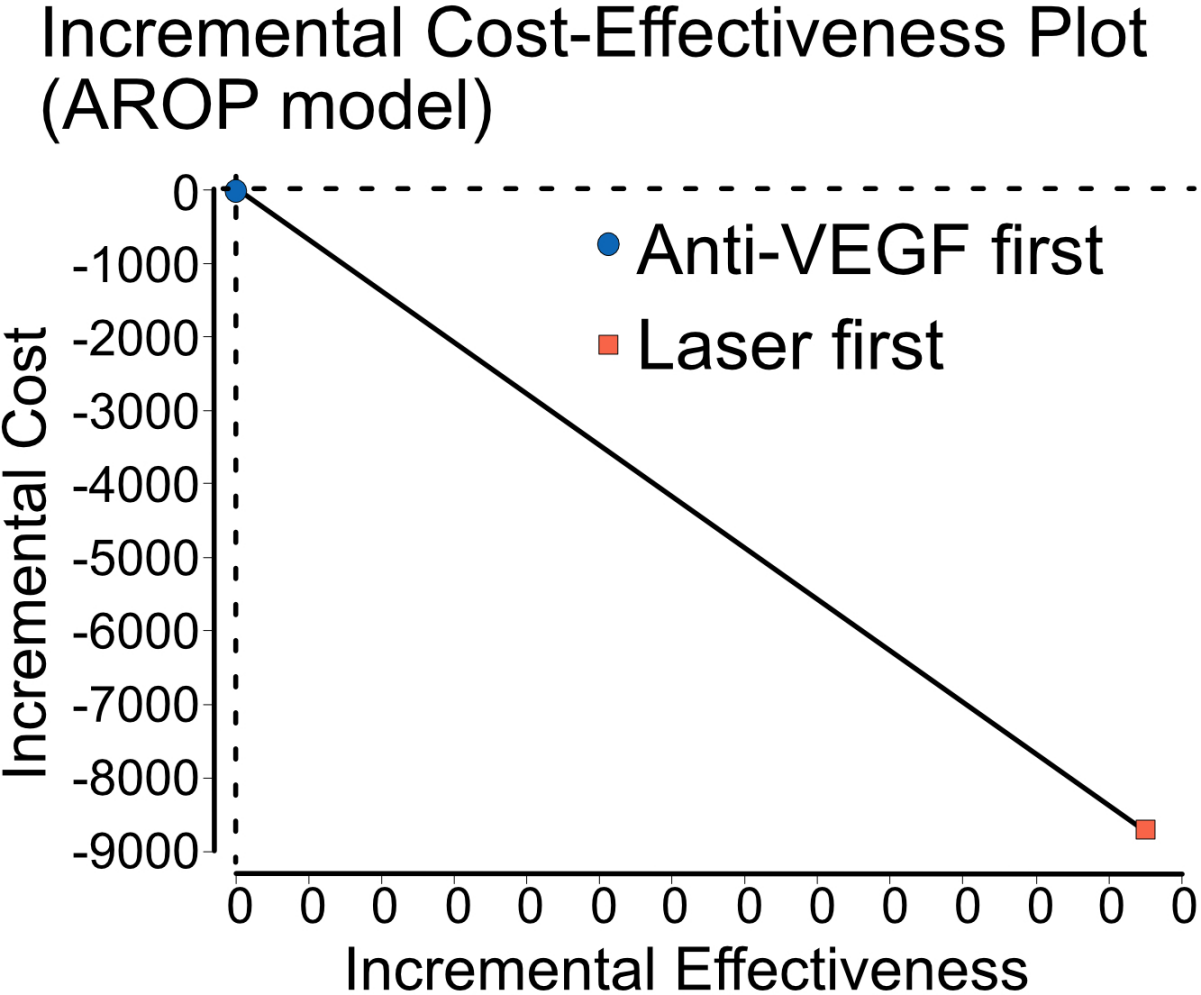
Cost-effectiveness plane comparing initial anti-VEGF and laser treatment among infants with aggressive posterior ROP (AROP). Costs for screening and treatment (Int$) and effectiveness based on vision outcomes derived from modelled real-world data. Anti-VEGF, anti-vascular endothelial growth factor.

To further explore whether any categorical factors could divide the population into groups with infants who had potentially received either of the treatments, the different treatment of infants with AROP between centres was identified as a possible candidate. We lacked detailed data on zone location (I vs II), precise age at treatment and anti-VEGF agent used (ranibizumab/bevacizumab/aflibercept), all of which may influence screening burden and retreatment. Although infants with AROP form a smaller subgroup, resulting in a small population for this model, some centres treated almost all infants with AROP with either anti-VEGF or laser therapy first (online supplemental table 2). These site-level differences in treatment patterns are consistent with previously reported national variation in ROP management^12^ and reflect real-world practice rather than balanced comparison groups. Limiting the model to only infants with AROP and incorporating the decision-tree model (figure 1), both results showed benefits for the infants initiating treatment with anti-VEGF, resulting in treatment initiation with anti-VEGF dominating the laser pathway (figure 2).

## Discussion

This study highlights that anti-VEGF treatment was associated with substantially higher screening costs and retreatment rates compared with laser, despite the lower direct cost of the anti-VEGF injection itself. However, this observation is complicated by selection bias and differences in clinical characteristics, including longer NICU stays in the anti-VEGF group. This illustrated the limitations of using observational data to create comparable groups for comparing treatment pathways and the challenges it poses for cost-effectiveness analysis, particularly when subgroups become smaller. Our retreatment rates were higher than those reported in RCTs and network meta-analyses,^11^ likely reflecting the extreme immaturity (<24 weeks GA) and centre-level practice patterns in our real-world cohort. A next methodological step would be to develop an NMA-informed decision-tree model using RCT-based effect estimates.

Recent studies have highlighted that treatment choice in ROP is context-dependent. A network meta-analysis showed lower retreatment rates with laser than with ranibizumab overall, while bevacizumab was associated with the most durable response in Zone I ROP.^11^ Other reviews suggest that anti-VEGF treatment may offer advantages in specific subgroups, although long-term outcomes and systemic safety require further evaluation.^10^ While anti-VEGF treatment offers advantages in specific subgroups, such as AROP, 5-year outcomes from the RAINBOW trial demonstrate comparable neurodevelopmental safety and visual outcomes to laser therapy, with no evidence of late reactivation-related complications.^19^ However, systemic safety concerns for bevacizumab—particularly neurodevelopmental effects—warrant ongoing evaluation.^20^

In our subgroup analysis of infants with AROP, where clinical equipoise may plausibly exist due to differences in regional treatment patterns, anti-VEGF is recommended according to national guidelines and emerged as a dominant strategy, associated with both lower costs and better outcomes. However, the AROP subgroup is small, and these findings should therefore be interpreted with caution. The limited sample reduces precision and limits the generalisability of the observed cost and outcome differences. Nevertheless, these findings underline the importance of identifying clinically meaningful subgroups where different treatment options can be considered equivalent, allowing for more reliable economic evaluation and potentially more efficient care. An additional consideration is the potential need for late re-treatment of persistent avascular retina after anti-VEGF, particularly in Zone I disease. Such risks contribute to increased screening and procedural burden and reflect the existing clinical equipoise regarding observation vs scheduled prophylactic laser after anti-VEGF injections. Further work is needed to better characterise these pathways and their resource implications.

### Strengths and limitations

The study’s main strength lies in its total population cohort, based on national registers with good coverage, and study-specific data collection from medical records, which have been validated for use in several studies. No bias was expected, as this population study uses national register sources with high validity. Moreover, the ROP screening threshold has changed over time; however, these changes do not affect this study population, but rather those born at 29 weeks’ gestational age. The main limitation is the cost estimation, as costs were identified from a single hospital, which may not be representative of costs nationwide. Additionally, the controllers providing these cost estimates acknowledged that they were estimates, as the actual costs for treatment and screening are not readily available in the hospital records. The reported estimates should be viewed as a first step towards future studies that cover all resource use in this population. Moreover, decision tree models are simple models, lacking a formalised time aspect, but in this study, the time is still indicated from the underlying data. While tree models are well-suited for comparing outcomes of alternative decisions, they can also be used to illustrate clinical pathways, even when no active clinical decision-making is involved. A full economic evaluation of ROP care should include long-term visual outcomes and societal costs. In our national cohort, ROP treatment was the strongest predictor of later visual impairment.^13^ These long-term consequences were not incorporated into the present acute-phase model but are highly relevant for future cost-effectiveness analyses.

### General discussion

This study presents a novel decision tree model that differs from previous health economic models in ROP, which have primarily focused on the presence or absence of treatment or the timing of screening.^7^ Instead, our model centres on the *initial treatment choice*—anti-VEGF versus laser—and maps clinical pathways and associated costs from a real-world, national cohort. This allows for the analysis of resource use beyond the direct treatment cost, capturing essential factors such as screening burden and NICU stay. While earlier models in ROP have explored screening intervals or thresholds for treatment, few have addressed the comparative cost-effectiveness of treatment modalities.

Cost-effectiveness modelling of anti-VEGF treatments has been more common in other retinal diseases such as diabetic retinopathy (DR), with simulation-based studies showing mixed results depending on setting and perspective. For example, a recent systematic review by Hodgson *et al* identified considerable uncertainty in the cost-effectiveness of anti-VEGF in DR, driven by drug costs and frequency of injections.^21^ However, these findings are not directly transferable to ROP, where the context, population and care pathways differ significantly.

To our knowledge, no previous cost-effectiveness analysis has compared initial treatment with anti-VEGF versus laser in ROP using real-world data. Our findings suggest that while anti-VEGF therapy is associated with higher overall costs due to increased screening and retreatments, in specific subgroups where clinical equipoise may exist—such as infants with AROP—it may be more cost-effective than laser therapy in terms of both cost and outcomes.

Our recent systematic review of ROP screening and treatment costs confirmed high variability across countries and care systems,^7^ underscoring the importance of locally derived data. The decision model we present contributes important context-specific evidence to support future evaluations and policy discussions in neonatal care.

### Implications

To use observational data in comparing health outcomes and resource use among preterm infants, a better understanding of the decision-making in initiating treatment is necessary. This calls for improved documentation in medical records and clinical registers, particularly in cases where new treatments are introduced. This study identified the challenges in comparing infants who initiate ROP treatment with either anti-VEGF or laser first. All models resulted in far higher costs among those initiating with anti-VEGF. However, we identified a small subgroup of patients for whom anti-VEGF became the dominant strategy, resulting in reduced costs and better health outcomes. However, it is challenging to generalise these findings to a larger population and draw conclusions on the cost-effectiveness of anti-VEGF.

## Conclusions

Putting the similarities in overall cost aside, the substantial difference in the number of screenings, especially in the anti-VEGF group, raises concerns about the efficiency and potential health effects of the numerous screening episodes. From a person-centred care perspective, this increased frequency is especially concerning for extremely preterm neonates, as it may elevate the risk of infection, increase patient burden and contribute to parental distress.

However, if identifying a (small) subgroup with expected clinical equipoise, anti-VEGF first was cost-effective and even dominated the laser pathway. The study’s findings will serve as a foundation for future register-based research investigating the long-term health and cost outcomes of this vulnerable patient population.

## Supplementary material

10.1136/bmjophth-2025-002507 online supplemental file 1

## Data Availability

Data may be obtained from a third party and are not publicly available. All data relevant to the study are included in the article or uploaded as supplementary information.
