# Supplementary material for: Modelling direct healthcare costs for screening and treatment of retinopathy of prematurity among infants born at gestational age <24 weeks: findings from a Swedish cohort
Source: BMJ Open Ophthalmol. 2025 Dec 31;10(1):e002507. doi: 10.1136/bmjophth-2025-002507 (PMC12766756; doi:10.1136/bmjophth-2025-002507)
Supplement: online supplemental file 1 [file bmjophth-10-1-s001.pdf]

Online only supplement:

Modelling direct healthcare costs for screening and treatment of Retinopathy of Prematurity among infants born at gestational age <24 weeks: findings from a Swedish cohort

Chatarina Löfqvist[1,2,3], Shambhavi Sharma[3], Ann Hellström[2], Hanna Gyllensten[1,3]

[1] Institute of Health and Care Sciences, Sahlgrenska Academy, University of Gothenburg, Gothenburg, Sweden.

[2] Department of Clinical Neuroscience, Institute of Neuroscience and Physiology, Sahlgrenska Academy, University of Gothenburg, Gothenburg, Sweden.

[3] University of Gothenburg Centre for Person-centred Care (GPCC), Sahlgrenska Academy, University of Gothenburg, Sweden.

Content

Figure S1 (A-D): Model iterations for ROP treatment pathways.

Table S1: Examinations and treatments, model A-B.

Table S2: Examinations and treatments, model AROP.

Figure S1 (A-D): Model iterations for ROP treatment pathways.

In the model, # represents the remaining probability needed to sum to 1 (or 100%) in a decision node, ensuring that all outcome branches are mutually exclusive and exhaustive (so if one branch has a probability of 0.436, the # would indicate the remaining probability of 0.564). Cost in (Int$).

A)

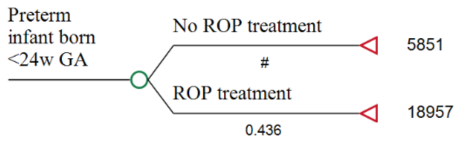

B)

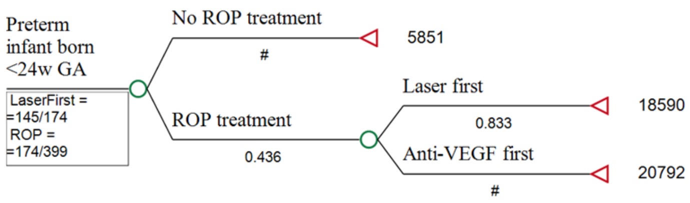

C)

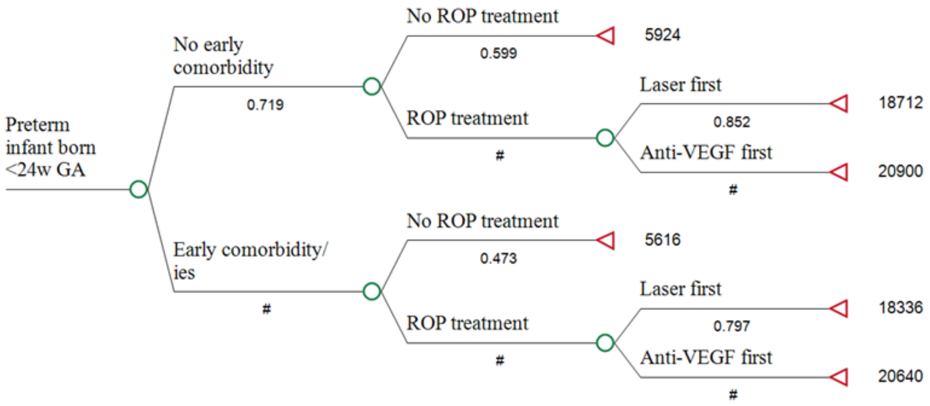

D)

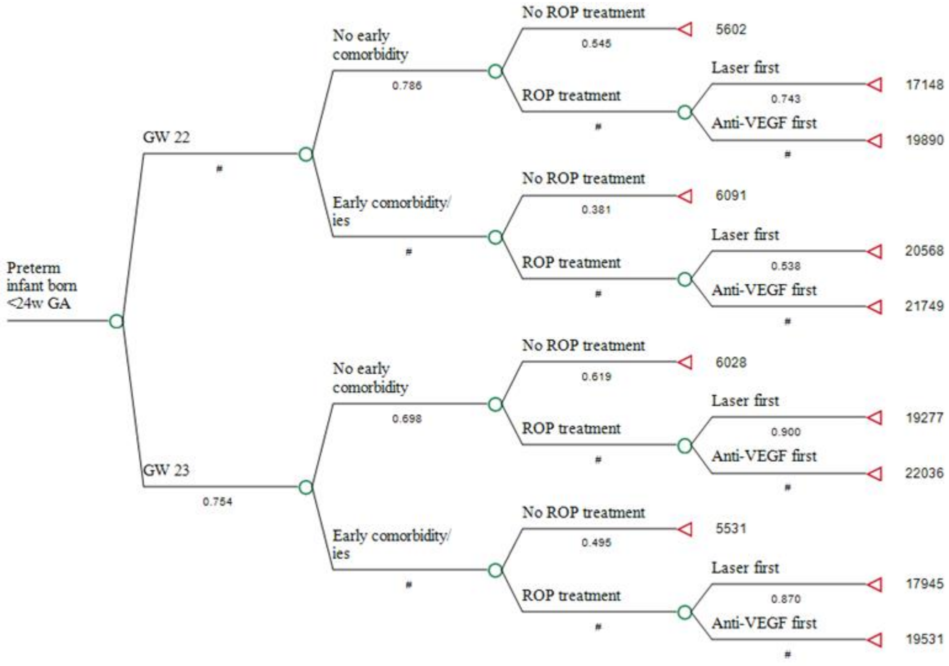
Preterm infant born <24w GA
GW 22
#
GW 23
0.754
No early comorbidity
0.786
Early comorbidity/ies
#
No early comorbidity
0.698
Early comorbidity/ies
#
No ROP treatment
0.545
ROP treatment
#
5602
Laser first
0.743
Anti-VEGF first
#
17148
19890
No ROP treatment
0.381
ROP treatment
#
6091
Laser first
0.538
Anti-VEGF first
#
20568
21749
No ROP treatment
0.619
ROP treatment
#
6028
Laser first
0.900
Anti-VEGF first
#
19277
22036
No ROP treatment
0.495
ROP treatment
#
5531
Laser first
0.870
Anti-VEGF first
#
17945
19531

Table S1: Examinations and treatments, model A-B.

Group	No ROP treatment	ROP treatment			Total
		Any treatment	*Laser first*	*Anti-VEGF first*	
	(n = 225)	(n = 174)	*(n = 145)*	*(n = 29)*	(N = 399)
	Mean [95% CI]	Mean [95% CI]	*Mean [95% CI]*	*Mean [95% CI]*	Mean [95% CI]
No. eye exams	13.3 [12.5–14.2]	17.6 [16.6-18.6]	*16.1 [15.1–17.1]*	*25.0 [23.0–26.9]*	15.2 [14.5–15.8]
No. laser sessions	N/A	1.2 [1.1-1.3]	*1.3 [1.2–1.4]*	*0.7 [0.5–0.9]*	0.5 [0.5–0.6]
No. anti-VEGF treatments	N/A	0.3 [0.2-0.4]	*0.2 [0.1–0.2]*	*1.2 [1.1–1.4]*	0.1 [0.1–0.2]
Days in Neo	152.9 [144.0–161.7]	155.9 [146.8–165.0]	*150.9 [141.4–160.4]*	*180.1 [154.8–205.3]*	154.2 [147.9–160.5]
Mean cost (Int$)	5851 [5485–6218]	18957 [17970-19944]	*18590 [17494–19686]*	*20792 [18628–22957]*	11567 [11091–12042]
Mean cost incl. NICU (Int$)	571946 [539101–604791]	596318 [562385-630251]	*577494 [542108–612880]*	*687749 [593617–781881]*	582837 [559308–606366]

Anti-VEGF, anti–vascular endothelial growth factor; CI, confidence interval; Int$, International dollar; Neo, neonatal care; NICU, Neonatal intensive care unit; No., number (of); ROP, retinopathy of prematurity.

Table S2: Examinations and treatments, model AROP.

Group	Laser first		Anti-VEGF first		
Sub-group	No Visual Impairment (n = 3)	Visual Impairment (n = 3)	No Visual Impairment (n = 10)	Visual Impairment (n = 6)	Total (N = 22)
	Mean [95% CI]	Mean [95% CI]	Mean [95% CI]	Mean [95% CI]	Mean [95% CI]
No. eye exams	21.0 [14.7–27.3]	28.7 [21.6–35.8]	23.7 [20.2–27.2]	28.0 [21.8–34.2]	25.2 [22.5–27.8]
No. laser sessions	1.3 [0.6–2.0]	2.0 [0.8–3.2]	0.7 [0.4–1.0]	0.5 [0.0–1.0]	0.9 [0.6–1.2]
No. anti-VEGF treatments	0.7 [-0.7–2.1]	2.0 [0.8–3.2]	1.3 [1.0–1.6]	1.2 [0.8–1.5]	1.3 [1.0–1.6]
Days in Neo	227.3 [110.4–344.3]	135.7 [112.5–158.8]	164.5 [129.1–199.9]	154.8 [99.5–210.2]	166.5 [139.1–193.9]
Mean cost (Int$)	22428 [10556–34299]	35545 [23585–47504]	20328 [16580–24077]	20130 [15733–24527]	22636 [19534–25737]
Mean cost incl. NICU (Int$)	864470 [426329–1302611]	538054 [460934–615174]	629636 [498359–760914]	593633 [387188–800078]	639352 [537317–741386]

Anti-VEGF, anti–vascular endothelial growth factor; CI, confidence interval; Int$, International dollar; Neo, neonatal care; NICU, Neonatal intensive care unit; No., number (of); ROP, retinopathy of prematurity.
